# “One time I fell, but I didn’t have to cry.” A qualitative study on everyday physical complaints in children

**DOI:** 10.1186/s12887-022-03442-8

**Published:** 2022-06-30

**Authors:** Sterre van der Ziel, Janna M. Gol, Michel J. van Vliet, Judith G. M. Rosmalen

**Affiliations:** 1 University of Groningen, University Medical Center Groningen, Interdisciplinary Centre of Psychopathology and Emotion Regulation, Groningen, The Netherlands; 2 University of Groningen, University Medical Center Groningen, Beatrix Children’s Hospital, Groningen, The Netherlands

**Keywords:** Symptom perception, Common-sense model, Illness behavior, Preschoolers

## Abstract

**Objective:**

Young children experience physical complaints, like abdominal pain or minor injuries from playing, almost every day. These experiences may shape how they deal with health issues later in life. While models exist to explain illness perception in adults, information is lacking on the perspective of young children. This qualitative study aimed to explore important themes in the experience of everyday physical complaints in four- and five-year-old children, using children as informants.

**Study design:**

30 semi-structured interviews were performed in which four- and five-year-old children were questioned about their experiences with everyday physical complaints. The interviews were double coded using Atlas.ti and subsequently qualitative content analysis was used to define themes.

**Results:**

All participating children were able to elaborate on their experiences with physical complaints. Three themes emerged from the interviews: causes of complaints, appraisal of complaints, and implications of complaints. In their appraisal of complaints, four- and five-year-old children made a distinction between visible and invisible complaints and real or pretended complaints.

**Conclusion:**

Four- and five-year-old children can already give details about their experiences with everyday physical complaints. They have developed ideas about the causes and implications of complaints and try to make an appraisal.

## Introduction

Illness perception is the organized way people think about their health conditions. This perception has a great influence on their emotional responses and adherence to management [1]. When researching symptom or illness perception, the Common-sense model (CSM) by Leventhal is widely used [2, 3]. This model consists of five domains of cognitive illness perception: identity, cause, time-line, consequences, and controllability or curability [2, 3]. Often the perception of illness starts with the perceived identity, naming the illness and its associated symptoms. Another important part of illness perception is the perceived cause of the illness. In the CSM, the other three dimensions are loosely associated, with consequences representing the perceived effect on life, time-line being the perceived time-frame for development and duration of illness, and curability or controllability being the perceived control on illness by the individual or others [2, 3]. While the CSM was developed in the context of chronic disease, it has also been found relevant in the context of common illnesses and perception of physical complaints in general [4].

The CSM is based on research on illness perception in adults. Although this perception is probably rooted in early life experiences, much less is known on illness perception and experience of symptoms in young children. Existing research in this age group often focuses on the experience of pain in the clinical setting, but information is lacking on the experience of physical complaints in general [5]. Young children experience small physical complaints or pains nearly every day [6, 7]. This could be everything from abdominal pain to small injuries from falling in the playground. These small everyday physical complaints might play an important role in the development of children’s responses to physical symptoms. The Social Learning Theory provides a theoretical framework to explain how experiences early in life may influence the development of illness perceptions [8]. This theory states that, in addition to learning by reinforcement and punishment, learning can also occur by observing behaviors and emotional reactions of others. As children observe their parents’ responses to physical symptoms from a young age, social learning might be particularly relevant for the development of illness perceptions [9]. Children thus learn from experiences with their physical complaints, but also by observing experiences of others having physical complaints [10]. This social context of learning starts at home in early life and is continued at school [8]. Insight into this learning process in young children might be relevant for the development of illness perception throughout life, but studies are scarce.

One of the reasons for this lack of knowledge is that young children are not considered reliable informants in scientific research. In most studies, either parents are asked about their children, or the children are observed rather than asked about their own views [6, 7, 11]. Yet, research shows that children can make reliable statements, provided the interview is adapted to their developmental level [12]. One study evaluated the dimensions of the CSM of illness perception in children by interviewing children at a primary school [13]. This study concluded that the CSM also fits the illness perception of these young children, confirming the existing model. However, the study started from the dimensions of illness perception identified in adults [14]. With an explorative design, the study might have identified different dimensions in young children. Some themes of illness perception included in the CSM might be less relevant for young children, as they are still in the pre-operational stage of development, as defined by Piaget [15]. This stage lasts from the age of 2 to the age of 7 and is characterized by the development of speech, egocentrism and concrete thinking [15]. The developmental stages of Piaget have been used in research on pain definition in young children [16]. It remains unknown which dimensions would emerge in explorative interviews with children on their perception of physical complaints and illness.

Our main objective of this study was to identify the important themes in the experience of everyday physical complaints using young children as informants. In addition, we aimed to explore how young children experience and describe physical complaints. Lastly, we aimed to compare the themes that emerge from interviews with children to the dimensions of the CSM [2]. To explore the children’s perspective, we used a qualitative research design to gain insight in their thoughts and experiences related to physical complaints. Young children were interviewed using a semi-structured interview guide. The results were subjected to a thematic content analysis to identify important themes in experiencing physical complaints. In this research we focused on four- and five-year-old children, as literature suggests they are the youngest age group with the ability to give information about their health status, and to give descriptions of experiences of illness [17, 18]. Furthermore, in the Netherlands, four- and five-year-olds start primary school. Thus their social context expands beyond their parents and siblings, providing us with the opportunity to include a broader social context in our study.

## Methods

### Participant selection and informed consent

The current study involving human participants was discussed by the Medical Ethics Review Board of the University Medical Center Groningen. The participants legal guardian provided written informed consent to participate in this study. A convenience sample was obtained with four- and five-year-old children from a primary school in a provincial town in the north of the Netherlands. This school was selected because their population of children was broadly representative for a typical school in this area of the country. We expected that recruitment via school would also reach parents who would not easily be interested in participating in scientific research. As the children from our sample went to a regular school, it can be assumed that they all had a normal development. To inform the parents, an information letter was placed on the digital environment of the school together with a short video in which the researcher explained the study. In the week after the parents were informed, the researcher was present at school every morning to provide information and to answer questions of parents. Informed consent was obtained from the parents of 31 out of 70 approached children. One of the 31 participants was thereafter excluded because of age, leaving a total sample of 12 four-year-old children (6 girls, 6 boys) and 18 five-year-old children (11 girls, 7 boys). Apart from age, no additional information was obtained on the children.

### The interview

Illustrations have been shown to facilitate interviewing young children by grabbing their attention and increasing their active participation, leading to more reliable results [19]. In order to attract the children's attention, to motivate them and to improve the conversation, we developed a picture book. Since the story had to be informal and neutral with a gender-neutral protagonist, we chose a story with animal characters. The book was about two dogs on their first day at school. Dog Iggy experiences an invisible complaint after waking up, namely abdominal pain. During the school day, Iggy also experiences a visible complaint: a bleeding knee after falling while playing during school break. The other dog, Ziggy, is Iggy’s friend and is wearing a band-aid. The pictures were used to ask questions following a semi-structured interview schedule. Both the interview and the pictures are accessible on the website of the Open Science Foundation [20].

We developed this interview guide using input from 10 adults working with young children in different contexts. We aimed to gather a varied ‘expert opinion’ and therefore included school teachers, sports teachers, family doctors and a psychologist. The interviewed experts were identified in the personal networks of the researchers. To obtain maximum variation with regard to professional background, we approached three additional experts via professional organizations. We used a semi-structured interview to ask these experts about their opinions and observations of important factors in young children’s experiences related to physical complaints. All the interviews were analyzed by open coding and the codes were then used to identify themes. These themes were used to create interview questions that matched the children’s perspective as closely as possible. In accordance with recommendations on qualitative interviewing of young children, we formulated open questions and avoided double questions [21].

The data collection was performed by SZ in January and February 2020, before the first case of COVID-19 was identified in the Netherlands. Before interviewing, SZ spent some days at the school to let the children feel comfortable with her presence. Before the interview, she told them that she was interested in learning from them. Children were interviewed individually in a familiar space in their school. They were motivated by the researcher to actively look at the pictures and answer the questions. The duration of the interview was approximately 25 min, but children were allowed to quit the interview at any time. All interviews were audio recorded and then verbatim transcribed. Data were analyzed after each set of three interviews, with the first set of three interviews serving as a pilot. After the pilot, the interview guide was critically evaluated and adjusted to better match the child's perspective or choice of words. This critical evaluation was continued after each set of three interviews. With this cyclical working method we aimed to get closer to the perspective of the investigated target group [22].

### Analysis

The analysis was carried out by SZ, a PhD student and medical doctor, who followed a course on qualitative research, and JG, a psychiatrist with experience in child and adolescent psychiatry and psychosomatic medicine. Besides her clinical work, JG works as a qualitative researcher and is trained and experienced in qualitative analysis. JR, researcher in the field of psychosomatic medicine, and MV, pediatrician, were regularly involved in the discussion of codes and themes and in the interpretation of results.

The analysis was performed using the conventional approach to content analysis [23]. All interviews were coded double-blind by SZ and JG, with no discussion before and during the coding. Coding was performed using Atlas.ti-8. Open coding was used for the first interview, after which the codes of the different coders were compared. When agreement was reached, the second transcript was coded, using the existing codes and supplementing them if necessary. After each coding session the final encoding of the transcript and the code list were discussed, and codes were renamed, merged and split. Twice during the coding process, all codes were discussed within the whole team. Few new codes were added after coding and discussing the first ten interviews, and the individual coding of both coders became increasingly similar. From that moment on, the coders coded two or three transcripts in a row before merging and discussing. Saturation was reached after coding 20 interviews, meaning that no new codes emerged when coding the last 10 interviews.

Before the analysis, fragments with quotes about the story or the characters were separated from fragments with statements about the children’s own experiences. In this way we aimed to diminish the influence of the storyline on the results. After coding all interviews, the outcomes were analyzed by One Sheet of Paper (OSOP) analysis, which is a method of visually rearranging the extracted data in order to define themes [24]. Summaries of all quotations belonging to a single code were written on a large sheet of paper and then rearranged based on differences and similarities. The results of the OSOP-method were discussed in the whole team, after which the final themes were defined. The entire coding and analyzing process was logged by the researcher to keep track of the thinking process and decisions that were made.

## Results

### General findings

All interviewed children actively participated in the interview and talked about at least one of their own experiences with physical complaints. Most of the interviewed children gave concrete answers to the questions, although there was a difference in the responses of four-year-olds and five-year-olds. Five-year-old children described social situations, feelings, and experiences more elaborately. Four-year-old children were more concrete and described less, jumped from one subject to another more often, and were generally more easily distracted. In spite of this, four-year-olds were more than able to tell about their own experiences concerning physical complaints, leading to roughly the same numbers of coded fragments as in the five-year-olds.

When children talked about complaints, their knowledge mostly seemed to be based on their own experiences or those of family members. When answering questions from the interviewer, they often spontaneously added that they had experienced the complaint themselves or that someone around them had experienced it. (Table 1, quote 1) School lessons or TV shows were hardly mentioned despite the health-themed school project they all participated in at the time. Their own experiences with complaints were mentioned in response to a question, spontaneously or in association with something that happened in the story about the dogs. However, in these associations many details were added that differed from the story. (Table 1, quote 2)

**Table 1:** Quotes general findings

**Interview quotes** (I: Interviewer; P: participant)	
Quote 1 – Boy, age 4	I: Yeah. When do you have to stay home from school? P: Um when he has a fever. I: When he has a fever. Yeah. And what- P: I once had a fever too. I: You once had a fever too? P: Yes, almost a whole day.
Quote 2 – Girl, age 5	I: Does he feel like eating something? P: Well, I don't think so with that tummy ache. I: No. Do you feel like eating something when you have a tummy ache? P: Well. Then I usually eat, my mommy makes me eat something, but then all I eat is a banana.
Quote 3 – Girl, age 5	I: His tummy. Yes. What's wrong with his tummy? P: He has a tummy ache. I: Yeah. How’s that? P: Maybe he's feeling poorly. I: Yeah. P: But I don't feel very well either. I: You don’t feel so well either? P: (quietly) No. I: What's bothering you? P: My tummy too. I: Your tummy too? Hmm, that's annoying. And how come- P: I've had it for a very long time.

A number of children commented once or more during the interview that they were currently experiencing complaints. Some of them casually showed the interviewer they had a scratch somewhere; others told the interviewer they had abdominal pain, sometimes adding they had had it for a very long time. (Table 1, quote 3)

### Theme 1: Causes of complaints

The first theme that emerged from the interviews was the causes of complaints (quotes related to theme 1 are in Table 2). Almost all children reported ideas about causes of abdominal pain and all children reported causes of visible complaints. When talking about causes of abdominal pain, most children mentioned a situation or possible behavior as cause of abdominal pain.

**Table 2:** Quotes theme 1 Causes of complaints

**Interview quotes** (I: Interviewer; P: participant)	
Quote 4 – Girl, age 5	I: Hmmm. But he’s got a bit of a tummy ache too. P: Yes. Because there’s a baby in his tummy. I: Yeah. Are there any other things that give you a tummy ache? P: Yes, if you eat lots of sugar. I: You can get a tummy ache from that. Is there anything else that could give you a tummy ache? P: Sweets. And if you eat a lot. And if you, um, run a lot then um, then um, then the tummy becomes s- um, um, um soft again. I: Yes. Yeah. Hey and did you ever have a tummy ache? P: Yes, very often.
Quote 5 – Boy, age 5	I: Yes. Hey and what could be wrong with Iggy's tummy? P: I don't know. I: No. If you have a tummy ache, why do you think that is? P: Maybe sickness or just that you have a little tummy ache. That you need to eat. Or drink.
Quote 6 – Boy, age 5	I: Hey and um, Iggy over here, has his first day of school today. Is it possible to get a tummy ache from that? P: (*nods head*) I: Yes? P: Because you're nervous about it.
Quote 7 – Boy, age 5	P: Then we went, I went to play soccer with my grandpa in the street. But then I fell over. I: Really? P: Because they, some tiles are higher than the others, so then I tripped over one. I: Hmm-mm. And then? P: Then I had blood on my knee. I: On your knee. And then what happened? P: Had to get a band-aid on it.
Quote 8 – Boy, age 5	P: But! I did have blood once, here. (*shows finger*) I: Yes, I see it! I can still see it on your finger! P: Yes, that was a very long time ago. That was because of ‘X’ (*name of peer*) I: What happened with ‘X’ then? P: No with me! I: Oh. What was there-. But what did it have to do with ‘X’? P: Well, went super fast on my bike AND he cut my finger, really badly!

Everyday reasons were more often suggested as cause for abdominal pain than illness-related causes. Children mentioned many own behaviors that might cause abdominal pain or had caused abdominal pain in the past, for example eating too much, too little, or bad food. (quote 4 & 5) A couple of children gave a disease-related cause for abdominal pain, such as ‘sickness’ or concussion. They did not further elaborate on these disease-related causes, but just mentioned the term. Very few children, all five-year-olds, mentioned something about stress or nerves causing abdominal pain. (quote 6)

When speaking of causes of visible complaints, all children mentioned a clear cause for injuries like bloody knees or scratches. Some children kept it simple by just saying that they fell, but many others were able to give more details about the circumstances. (quote 7) Some children added that falling on a stone pavement or playground caused their injury. A few children mentioned a culprit caused their visible injury, such as a peer or a sibling. (quote 8)

### Theme 2: Appraisal of complaints

The second theme that emerged from the data was the appraisal of complaints (quotes are in Table 3). This appraisal can be divided into three different dimensions: visible versus invisible complaints, real versus pretended complaints, and estimating the severity of complaints. The first dimension is whether or not the complaint is visible. When talking of visible complaints and small injuries, all children had clear ideas of the concept of the complaint. Visual complaints seemed to be appraised by the presence of blood, and blood or bleeding appeared to be a theme when talking about complaints. Injuries causing bleeding were often described in many superlatives. (quote 9) When talking of more internal, invisible pain, children mainly talked about abdominal pain and less often about headache or earache. Children seemed to have more difficulty appraising headache or earache and often made a short statement, followed by “I don’t know” when the interviewer asked follow-up questions. Some children did elaborate on the visible aspects of their internal complaint, such as throwing up or having diarrhea.

**Table 3:** Quotes theme 2 Appraisal of complaints

**Interview quotes** (I: Interviewer; P: participant)	
Quote 9 – Boy, age 5	P: Um… Then I had a lot of blood. I: Really? P: Yes. I: Yes? And, um, how did you get a lot of blood? P: I had fallen really badly.
Quote 10 – Girl, age 5	I: Is it also possible that he is pretending to have a tummy ache? P: Hmm-mm. I: That he doesn’t really have one? P: Yes, I think he is. Because he's very much into eating now. So it may have gone away or he's not really sick. Or he's just kidding.
Quote 11 – Girl, age 5	I: Yes. And could it also be that he is pretending to have a tummy ache? P: Yes. I: Yes? P: Because he just doesn't feel like going to school. I: Yes. And do you know anyone who does that? P: Hmmmm… Yes, my brother, sometimes he doesn't want to go to school. And then he just starts pretending.
Quote 12 – Boy, age 5	I: Yes. And did that hurt too? P: Well. I think it did, because I did cry for a long time.
Quote 13 – Girl, age 4	I: (…) Do you also know when you have to go to the doctor? P: (nods head) I: When is that? P: When you are really hurting super bad.

A second dimension that was identified in the children’s appraisal of complaints was their estimation of whether or not a complaint was real. The interview guide contained a question on the possibility that a physical complaint was not real, but pretense. All children were able to understand this question. (quote 10) However, this theme was also mentioned spontaneously by several children. When speaking of the pretense of physical complaints, many indicated that it is actually not allowed. Some children elaborated on peers or siblings that they observed while feigning complaints, but firmly stated they would not do such a thing. Those peers were often described as naughty. Main reasons children gave for pretending complaints were joking, fantasy play, or a way to get what you want. (quote 11) Most children indicated that their parents did not believe in their pretended complaints or that parents might even get angry. Besides elaborating on these pretended complaints, children also spoke of strategies to distinguish real complaints from pretending, for example, crying was reported as a sign that a complaint was real.

Crying was also sometimes mentioned as measure of the severity of a complaint (quote 12), the third dimension concerning the children’s appraisal of complaints. A few children mentioned the use of medication as a hint that their complaint was severe. This appraisal of severity was sometimes mentioned spontaneously, but also in response to a question. For example, all children were asked about an indication to visit a doctor, but some had already mentioned this spontaneously. Reasons for going to the doctor were abdominal pain, earache, broken legs or arms, or being sick. Several children seemed to use superlatives to magnify complaints that needed a doctor. (quote 13).

### Theme 3: Implications of complaints.

The third theme that was extracted from the interview data concerns implications of complaints (quotes are in Table 4). In response to the question what the children would do when experiencing complaints, the majority of children first responded that they would tell their parents or their teacher; only a few mentioned that they would keep quiet about their complaints. When recalling what they told their parents or teacher, they mentioned a very short description of their complaint. (quote 14) The children described the reaction of the adults as giving practical solutions such as a band-aid or a glass of water. They rarely talked about receiving (or needing) physical or emotional comfort in response to physical complaints.

**Table 4:** Quotes theme 3 Implications of complaints

**Interview quotes** (I: Interviewer; P: participant)	
Quote 14 – Girl, age 4	I: No. Do you tell your mommy when you have a tummy ache? P: Hmm-mm. Yes. I: Yes? What do you say? P: Then I say, mommy I have a tummy ache. I: And then what does mommy say? P: Then you just have to go to the doctor, mommy said.
Quote 15 – Girl, age 5	I: (…) Does a band-aid help with the blood? P: (shakes head) I: No? P: Cream works better.
Quote 16 – Boy, age 5	I: No. How long does it last, when you have blood? P: Um, one night and then it's gone already.
Quote 17 – Girl, age 5	I: Yes? So where did it hurt at that time? P: By my tummy. I: By your tummy too. Just like Iggy. And what did you do then? P: Then I snuggled up on the couch, watching Youtube with songs.

Besides seeking support from an adult, the implications of complaints can be divided into solutions and consequences. Children had a lot of ideas about suitable behaviors as a solution to their complaints. For example, many children suggested taking it easy and lie in bed or on the couch when having complaints, especially when experiencing abdominal pain or sickness. Likewise, a lot of children specified their ideas about what to eat and what not to eat when having abdominal pain. A few children spontaneously mentioned medication as a solution to their complaints. Some children elaborated on the effect of a remedy, such as medication or lying on the couch. (quote 15)

When it comes to consequences of complaints, children had different ideas about playing when having physical complains. Some children felt that abdominal pain was a reason not to play, but to stay inside and rest. Others indicated to play more calmly, or reported no effect of the complaint on playing. A few children felt they could not play with a band-aid. All children expressed ideas about going to school with physical complaints and reasons to stay home from school. Frequently mentioned reasons for staying home were being sick, having a fever, vomiting or (abdominal) pain. Many children made the remark that, in order to stay home, the complaints had to be real, as mentioned in the second dimension of theme two. Only a few children indicated that they went to and stayed at school despite having complaints.

Children reported little about the timeline of complaints. All children were asked about the time it takes for a complaint to pass, but less than half of the children could answer this question. Some seemed to randomly name a time unit or number, while others stated that it takes a night’s sleep for a complaint to pass. Children did not report doubts on whether the complaints would pass. (quote 16)

Only one child spoke of being sad, as a consequence of his complaint. Besides that, negative emotions such as sadness or fear were hardly mentioned. However, many children mentioned the advantages or positive sides of having a complaint, such as being allowed to watch TV, getting an ice-cream or sleeping in their parents’ bed. (quote 17)

## Discussion

We showed that four- and five-year-old children were able to give details about their experiences with everyday physical complaints. They have developed ideas about the causes and implications of complaints and try to make an appraisal. Their knowledge seemed to be based on their own experiences. Their ideas about causes were more often related to normal bodily functions or their own actions than to disease. For the appraisal of complaints, children used visible aspects such as bleeding and diarrhea and crying to assess the severity of the complaint and whether it is real or pretense. Children mentioned several solutions of complaints, but their initial response seemed to be seeking support from adults. When speaking of consequences, they focused more on the advantages of having complaints than on the disadvantages.

Previous research showed that preschool-aged children can already talk about pain and are able to describe it. They do this in a concrete way without mentioning feelings or sentimental descriptions of their experience [25–28]. Our results confirmed this factual description of complaints, with almost no children explicitly talking about their emotions related to complaints. This can be explained by their developmental stage, as defined by Piaget [15, 16]. Four- and five-year-old children are still in the so-called preoperational phase, which assumes that children have not yet developed thinking on an abstract level. Their developmental stage might also be the reason that the children in our study were more elaborate in their description of visible complaints, compared to internal complaints, as internal complaints might be more abstract [15].

However, almost all children were able to mention both internal and external causes for their complaints. This knowledge on causality of illness and symptoms is in accordance with previous research [29]. Mentioning own behaviors as a cause for internal and external complaints and linking food to abdominal pain has been reported in earlier studies [30]. In contrast, other studies reported that preschool-aged children mostly state that pain is caused by external injuries or that it occurs spontaneously [25]. Yet, those reports resulted from quantitative data, which may be the explanation for the discrepancy with our findings.

Our results show that children as young as four years old know that physical complaints can be feigned, can give examples of this and can tell how their parents react. Some mentioned that they would quickly tell that they were joking upon noticing that their parents did not believe their complaint. To our knowledge, it has never been described before that children themselves make a distinction between physical complaints that are real and physical complaints that are pretense. There is only one study mentioning children pretending to have symptoms, but these results were based on parents’ observations of their young children using symptoms instrumentally, for example, to gain attention [31]. Most importantly, this pretending of complaints can be considered part of normal development of cognition and moral reasoning [32].

In line with previous studies, we found that children of this age already see an active role for themselves in reducing pain or complaints, and also have ideas about strategies for pain relief [33]. However, very little research exists on children’s coping with *everyday* physical complaints, compared to research in the clinical setting. We showed that children already have their own ideas about how they can alleviate symptoms. Seeking social support seemed to be the first strategy of children, emphasizing the importance of the response of adults and social learning processes. These social learning processes can also be linked to our result that children talk mainly of their own experiences and of those close to them. The behaviors and cognitions that are described by the children give a reflection of their social context and how they may have learned about physical complaints [9, 10]. Our study suggests that social learning develops in a broad social context, as some children share experiences of peers having complaints, such as a classmate throwing up or a peer hurting them on the playground. In contrast, although all children participated in a health-themed school project at the time of the interviews, none of them referred to these project activities. This raises the question whether social learning might be more relevant than direct instruction for the development of illness perceptions in young children.

When comparing our results to the dimensions of the Common-sense model, we are not able to conclude that all dimensions fit the children’s perspective, contradicting earlier research [2, 13]. Dimensions such as identity, causality and consequences of complaints are also found in children, but this is not true for the dimensions time-line and controllability. Only a few children elaborated on the time-line of their complaints and even fewer seemed to be able to give an accurate description of this time-line. When it comes to curability or controllability, it is more difficult to interpret if this is a theme or dimension for children. They do not seem to have any doubt that their physical complaints will pass and that their parents or teachers will have a solution.

### Strengths and limitations

To the knowledge of the researchers, no previous qualitative research exists in which children themselves were interviewed on how they experience everyday symptoms. In this study we captured the perspective of children and treated the children as valuable informants [34]. This included taking into account a variety of factors such as a safe location for the interview, an appropriate interview format, and becoming familiar with the researcher's presence in school beforehand. The explorative design is another strong aspect of this study, as this adds to other interview studies on the subject of health and symptoms in this age group that did not have an explorative design [7, 29, 35]. Another strength of this study is its thoroughness in methodology and documentation, which is an important aspect of qualitative research. Our research was conducted in line with the Consolidated criteria for Reporting Qualitative Research (COREQ) checklist [36]. For insight in our methodology, we uploaded our logbook and the completed checklist to the website of the Open Science Foundation [20]. A further strength to this study is our multidisciplinary team, consisting of expertise in different fields: both clinical and non-clinical, in pediatrics, psychiatry and psychosomatic medicine. The difference in expertise made it possible to unravel more layers in our discussions during the analysis. With regards to the data collection, this was mostly executed by SZ who was trained, but had not conducted qualitative research before. Therefore she was intensively supervised by JG, an experienced qualitative researcher who closely monitored the data collection.

This study also has limitations. This research was conducted to capture the perspective of children. Therefore, an effort was made to focus on this as much as possible and to let the children’s perspective guide the data collection. Despite the fact that the researcher and supervisors were aware of their 'adult' way of looking and interpreted as little as possible, their prior knowledge might have colored their view to some extent.

Secondly, as our interview questions were based on a picture book, the story could have influenced the children’s answers. However, we coded their interpretations of the pictures and expectations of the story separately from their own experiences with complaints. In the subsequent analysis, little correlation was found between the story and their own experiences of complaints.

Thirdly, we aimed finding participants in a school that was in an average Dutch neighborhood. We succeeded regarding the socio-economic status of the families, but failed regarding cultural diversity. It is possible that the results would have been different if the study had been done at a school in a multicultural neighborhood. Moreover, as we aimed to define the experience of children in general, we do not know if these experiences are generalizable to children with chronic health problems. No additional information about health status of the children or their parents could be obtained, so we were not able to analyze whether family experiences related to disease influenced the perceptions of the children. Although we regard this a limitation of our study, we focused on everyday complaints and assumed a normal variation of health status in our sample. Additionally, the lack of questionnaires on health status in children and parents may have resulted in a lower barrier for parents to consent to participation.

### Implications for research and practice

Little research has been carried out regarding the experience of physical complaints in children, but our study makes clear that these experiences already show individual differences. Further insight into these individual differences might be useful since early experiences related to the body and illness may influence coping with symptoms later in life. To study whether differences in everyday complaints are informative to detect children at risk for high symptom levels, a longitudinal study in a large sample of young children is essential. This implies that new research methods are needed that are able to assess children’s experiences with everyday complaints in large samples. Previous studies have used the Berkeley Puppet Interview in this age category, but this interview does not include questions about illness perception. Our study provides themes on which such questions could be based to match the children’s perspective concerning experiences with physical complaints. To further investigate the role of social learning processes, it would be important to include the parents’ perspectives on illness perception as well. Longitudinal data collected in both parents and children will give more insight in the development of illness perceptions from early childhood, and the consequences for coping with symptoms later in life.

In medical practice the results of this study could enable practitioners to better connect with the perspective of the young child, instead of using the adult perspective. Four- and five-year-olds already have developed many ideas about their complaints and should therefore be involved in the conversation about their complaints. Children can be involved while taking their medical history, and also in the management of their complaints. When educating parents and demystifying the complaints, physicians should strive to involve the child in the conversation. In this way, the physician can start the prevention of the mystification of physical complaints in children from a young age.

## Conclusions

Our study showed that four- and five-year-old children can already give details about their experiences with everyday physical complaints. They have developed ideas about the causes and implications of complaints and try to make an appraisal of the severity. While these themes match some dimensions of the CSM, particularly identity, cause, and consequences, other dimensions such as time-line and controllability seem less relevant in this age-group. Their own experiences seem to play an important role in their knowledge and ideas about physical complaints.

## Data Availability

The datasets used and/or analyzed during the current study are available from the corresponding author on reasonable request.

## Abbreviations

CSM: Common-sense model
OSOP: One Sheet of Paper
